# Effect of Microencapsulated Fermented Catfish Waste Extract on Apparent Metabolizable Energy, Nutrient Digestibility, Digestive Enzyme Activity, and Intestinal Microbiota of Broiler Chickens

**DOI:** 10.1002/fsn3.72012

**Published:** 2026-06-10

**Authors:** Abun Abun, Kiki Haetami, Rahmad Fany Ramdhan

**Affiliations:** ^1^ Department of Animal Nutrition and Feed Technology Padjadjaran University Sumedang West Java Indonesia; ^2^ Department of Fisheries Padjadjaran University Sumedang West Java Indonesia

**Keywords:** antibiotic alternative, broiler, catfish processing waste, fermentation, maltodextrin, microencapsulation

## Abstract

Microencapsulated fermented catfish waste extract (MFCWE) is a promising, circular‐economy feed additive that may help replace antibiotic growth promoters while valorizing catfish‐processing byproducts through fermentation and microencapsulation. This study evaluated graded dietary inclusion levels of MFCWE (0.5%–2.0%) on energy utilization, nutrient digestibility, digestive enzyme activity, and selected intestinal microbes in broilers. A completely randomized design was used with six treatments: basal diet (T‐), basal diet + zinc bacitracin (100 ppm; T+), and basal diet supplemented with 0.5%, 1.0%, 1.5%, or 2.0% MFCWE (T0.5–T2). At day 35, birds were subjected to a metabolizable energy trial and sampling for digestibility, enzyme activity, and microbial counts. MFCWE affected AME (*p* = 0.003), AMEn (*p* = 0.007), and nitrogen retention (*p* < 0.001), with the highest AME/AMEn values observed at 1.5%–2.0% inclusion and the highest nitrogen retention at 1.5%. Dry matter and organic matter digestibility were highest at 1.5% MFCWE, representing increases of 8.6% and 7.6%, respectively, relative to T‐ (both *p* < 0.001), and were comparable to T+. Amylase activity increased with MFCWE (*p* = 0.005), while *Staphylococcus aureus* counts were reduced by 16.6% at 1.5% inclusion relative to T‐ (*p* = 0.003); total aerobic bacteria and *Escherichia coli* were not affected (*p* > 0.05). Overall, 1.5% MFCWE provided the most consistent improvements across endpoints, supporting its potential as a functional, microencapsulated feed additive and a value‐added route for catfish‐processing waste utilization.

## Graphical abstract

Cell‐free fermented catfish‐processing waste was microencapsulated with maltodextrin to produce MFCWE, a circular‐economy feed additive for broiler chickens. Dietary inclusion at 1.5% produced the most consistent overall response, improving apparent metabolizable energy, dry matter and organic matter digestibility, intestinal amylase activity, and reducing *Staphylococcus aureus* counts while supporting the valorization of fish‐processing byproducts.

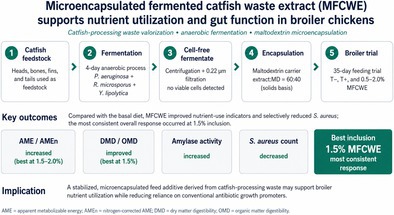

## 1 Introduction

Broiler production continues to expand to meet increasing demand for affordable animal protein. Historically, antibiotic growth promoters (AGPs) were widely used to stabilize gut health and improve feed efficiency; however, concerns about antimicrobial resistance and residue stewardship have accelerated the search for effective alternatives. Postbiotics (i.e., preparations of nonviable microorganisms and/or their components and metabolites) are increasingly explored because they can remain stable during feed processing, are less sensitive to storage conditions than live probiotics, and can modulate gut function and pathogen pressure (Ayalew et al. 2022; Kour et al. 2025; Saeed et al. 2023; Zhu et al. 2021).

Fish processing byproducts represent an abundant and underutilized resource rich in protein and lipids. Converting these byproducts into value‐added feed ingredients can reduce environmental burdens while supporting a circular bioeconomy. Catfish processing waste (e.g., heads, bones, fins, and tails) is generated in large quantities and is often poorly utilized, despite its potential as a source of functional peptides and lipids (Jasrotia et al. 2024; Murugan et al. 2024; Patil et al. 2024; Sivaranjani et al. 2024).

Microbial fermentation can increase the functional value of fish waste by hydrolyzing macromolecules, releasing peptides and free amino acids, and generating microbial metabolites with potential antimicrobial and antioxidant properties. In the present study, a synergistic consortium comprising a bacterium ( *Pseudomonas aeruginosa* ), a mold (*Rhizopus microsporus*), and a yeast (*Yarrowia lipolytica*) was applied to promote proteolysis and lipolysis during fermentation, followed by sterile filtration to obtain a cell‐free fermentate (Abun, Haetami, et al. 2025; Abun, Rusmana, et al. 2025; Pham et al. 2022).

Nevertheless, fermented fish extracts are prone to oxidation and quality deterioration. Compared with non‐encapsulated fermented products, microencapsulation using maltodextrin‐based carrier systems (alone or blended with gum Arabic or other biopolymers) can better protect sensitive compounds from oxygen and moisture, improve powder handling and storage stability, facilitate more homogeneous feed inclusion, and potentially modulate the release of functional components along the gastrointestinal tract (Cui et al. 2023; de Souza et al. 2023; Mirzabagherian and Noorbakhsh 2025; Sirichokworrakit et al. 2025).

Despite increasing interest in postbiotics and fermented byproducts, evidence remains limited regarding the physiological impact of microencapsulated, cell‐free fermented catfish waste extract on energy utilization, nutrient digestibility, digestive enzymes, and intestinal microbial indicators in broilers under practical feeding conditions (Brito et al. 2020; Dastar et al. 2025; Rybicka et al. 2024; Woyengo et al. 2025).

Therefore, this study evaluated graded dietary levels of microencapsulated fermented catfish waste extract (MFCWE; 0.5%–2.0%) in broiler diets compared with a basal diet without zinc bacitracin (negative control) and zinc bacitracin supplementation (positive control). We hypothesized that, as a microencapsulated postbiotic‐like preparation (cell‐free fermentate), MFCWE would (i) improve apparent metabolizable energy and nitrogen retention, (ii) enhance apparent digestibility of nutrients, (iii) support digestive enzyme activity, and (iv) selectively suppress opportunistic/pathogenic bacteria via fermentation‐derived organic acids and bioactive peptides, with an optimal response around 1.5% inclusion (Beckman et al. 2024; Yasmeen and Ahmad 2025). To our knowledge, this is among the first studies to integrate food‐waste valorization, microbial fermentation, and low‐technology hot‐air microencapsulation of a cell‐free catfish waste fermentate in a broiler feeding trial spanning metabolizable energy, nutrient digestibility, digestive enzyme activity, and intestinal microbial indicators.

## 2 Materials and Methods

### 2.1 Ethics and Animal Welfare

All animal procedures were approved by the Research Ethics Committee of Universitas Padjadjaran, Bandung, Indonesia (Protocol No. 231/UN6.KEP/EC/2025; approved on 14 March 2025). Husbandry and handling were conducted to minimize stress and suffering, and the number of animals used was limited to the minimum required for statistical power. This study is reported in accordance with ARRIVE 2.0 (Essential 10).

### 2.2 Experimental Design and Birds

A completely randomized design was implemented with six dietary treatments and four replicate pens per treatment (6 treatments × 4 pens × 10 birds = 240 birds). The feeding trial lasted 35 days. Day‐old Cobb‐500 broiler chicks (*n* = 240; mixed sex) with an initial body weight of 45.30 ± 3.37 g were randomly allocated to 24 pens (10 birds/pen). Feed and water were provided ad libitum throughout the study. No vaccination program was applied during the trial because the DOCs had already been vaccinated by the breeder, and no additional medication was administered during the feeding trial. Ambient house temperature was maintained at 33°C–35°C (days 1–7), 28°C–32°C (days 8–14), 25°C–29°C (days 15–21), 22°C–24°C (days 22–28), and 22°C–25°C (days 29–35), with corresponding relative humidity ranges of 60%–65%, 55%–60%, 50%–55%, 50%–55%, and 50%–55%, respectively. Descriptive temperature–humidity index (THI) values, calculated from ambient temperature and RH, ranged from 30.7–32.8, 26.1–29.8, 23.4–27.0, 20.8–22.7, and 20.8–23.5 across the same periods.

Treatments consisted of a basal diet without zinc bacitracin (T−; negative control), a basal diet supplemented with zinc bacitracin (100 ppm; T+; positive control), and a basal diet supplemented with MFCWE at 0.5%, 1.0%, 1.5%, or 2.0% (T0.5, T1, T1.5, and T2). At day 35, two birds per pen were randomly selected for metabolizable energy determination and subsequent sampling for digestibility, enzyme assays, and microbial enumeration. For each endpoint, measurements from birds within the same pen were averaged to obtain a single pen value. The selected experimental size was based on previous comparable broiler feeding studies, available pen capacity, and practical/statistical considerations for maintaining four replicate pens per treatment.

### 2.3 Preparation of Fermented Catfish Waste Extract

Catfish processing waste (head, bones, fins, and tails) was ground and fermented anaerobically for 4 days at 35°C. The microbial consortium consisted of *P. aeruginosa, R. microsporus*, and *Y. lipolytica* with an inoculum dose of 10% (v/w). During fermentation, pH was monitored daily using a HI98100 Checker Plus pH tester (Hanna Instruments, Woonsocket, RI, USA), while temperature and moisture were also recorded. Total titratable acidity (TTA) was quantified by titration with 0.1 N NaOH to pH 8.2 and expressed as g lactic acid/L. Soluble protein/peptide content was quantified by the Bradford method using Coomassie Brilliant Blue and a protein standard curve, with absorbance read using a SpectraMax 190 microplate reader (Molecular Devices, Sunnyvale, CA, USA). Volatile fatty acids (VFAs; acetate, propionate, and butyrate) were analyzed by GC‐FID using a Varian CP‐3380 gas chromatograph equipped with an SP‐2340 column (Varian, Mississauga, Ontario, Canada) and an internal standard, but were not detected (below the laboratory‐reported LOD/LOQ (mM (mmol/L)): acetate 1.63, propionate 0.16, butyrate 0.067).

### 2.4 Biosafety Risk Management for *Pseudomonas aeruginosa*

Because *P. aeruginosa* is an opportunistic pathogen, all culturing and fermentation steps were performed under biosafety level 2 (BSL‐2) conditions with appropriate containment, disinfection, and waste management. To ensure that the final product functioned as a cell‐free fermentate, the supernatant obtained after fermentation and centrifugation (Vet PRP compact centrifuge, Cell Therapy Sciences, Coventry, United Kingdom) was sterile‐filtered (0.22 μm) before microencapsulation. Sterility verification was performed by plating the filtrate and reconstituted microcapsules onto cetrimide agar (selective for *Pseudomonas* spp.) and tryptic soy agar; no viable colonies were detected after 48 h incubation at 37°C. Based on the spread‐plating volume of 0.1 mL without enrichment, the analytical limit of detection was 10 CFU/mL; the results were reported as ‘no viable cells detected’ at the assay LOD.

### 2.5 Microencapsulation Procedure and Quality Control

The cell‐free fermented extract was mixed with maltodextrin (MD) as a wall material to a final extract: MD ratio of 60:40 (w/w, solids basis). This ratio was selected based on preliminary laboratory trials, in which it provided the most suitable drying behavior and final powder characteristics for the catfish waste extract. The mixture was homogenized to achieve a uniform slurry using a commercial heavy‐duty blender (Crown Horeca SC‐X385) and dried in a forced‐air oven (GOYOJO Lab Forced Air Convection Drying Oven, 43 L; GOYOJO, Kwun Tong, Hong Kong) at 50°C to constant mass, then milled and sieved through a 180‐μm mesh to obtain free‐flowing microcapsules (MFCWE). Product yield was calculated as the mass of dried microcapsules relative to total solids input. Moisture content was determined gravimetrically by oven‐drying to constant weight. Water activity (aw) was measured at 25°C using an AquaLab water activity meter (Meter Group, USA) after sample equilibration. Encapsulation efficiency (EE, %) for protein/peptide was calculated as EE = (T—S)/T × 100, where T is total protein/peptide in microcapsules after complete dissolution, and S is surface protein/peptide recovered by a brief aqueous wash; protein/peptide concentrations were quantified using the Bradford assay. Particle size was standardized by sieve cut‐off (180 μm); therefore, D50 is reported as an approximate sieve‐based indicator (≤ 180 μm). Proximate composition (crude protein and ether extract) was determined for product documentation.

### 2.6 Diet Formulation

The ingredient composition and analyzed nutrient composition of the basal diet are shown in Table 1. Yellow corn, soybean meal, rice bran, fish meal, bone meal, lysine, and methionine were analyzed before formulation, and the nutrient composition of the basal diet was determined by a commercial laboratory. Therefore, the nutrient values reported in Table 1 represent the analyzed nutrient composition of the basal diet rather than calculated values. A single basal formula was used throughout the 35‐day feeding period. The experimental treatments are summarized in Table 2. Top mix was included at 0.5 kg/100 kg diet as a commercial vitamin‐mineral‐amino acid premix and did not contain zinc bacitracin. Zinc bacitracin was added only to the positive control diet (T+) at 100 ppm.

**TABLE 1:** Ingredient composition and analyzed nutrient composition of the basal diet.

Item	Amount
Ingredients (kg/100 kg)	
Yellow corn	53
Soybean meal	26.35
Rice bran	9
Fish meal	6.75
Bone meal	2
CaCO_3_	0.5
Coconut oil	1.5
Lysine	0.15
Methionine	0.25
Top mix	0.5
Nutrient composition	
Metabolizable energy (kcal/kg)	2826
Crude protein (%)	21.25
Crude fiber (%)	2.71
Ash content (%)	9.08
Extract ether (%)	4.87
Calcium (%)	0.95
Phosphorus (%)	0.68

*Note:* Ingredient inclusion is expressed as kg/100 kg diet. Top mix was included at 0.5 kg/100 kg diet as a commercial vitamin‐mineral‐amino acid premix and did not contain zinc bacitracin; its manufacturer‐declared composition is provided in Section 2.6.

**TABLE 2:** Dietary treatments used in the study.

Treatment	Description
T−	Basal diet without zinc bacitracin (negative control)
T+	Basal diet + zinc bacitracin (100 ppm; positive control)
T0.5%	Basal diet + 0.5% MFCWE
T1%	Basal diet + 1.0% MFCWE
T1.5%	Basal diet + 1.5% MFCWE
T2%	Basal diet + 2.0% MFCWE

According to the manufacturer‐declared composition, each 10 kg of Top mix contained vitamin A, 12,000,000 IU; vitamin D3, 2000,000 IU; vitamin E, 8000 IU; vitamin K, 2000 mg; vitamin B1, 2000 mg; vitamin B2, 5000 mg; vitamin B6, 500 mg; vitamin B12, 12,000 mg; vitamin C, 25,000 mg; calcium‐D‐pantothenate, 6000 mg; niacin, 40,000 mg; choline chloride, 10,000 mg; methionine, 30,000 mg; lysine, 30,000 mg; manganese, 120,000 mg; iron, 20,000 mg; iodine, 200 mg; zinc, 100,000 mg; cobalt, 200 mg; and copper, 4000 mg. Zinc bacitracin was not detected in the premix.

### 2.7 Metabolizable Energy and Nitrogen Retention

At day 35, selected birds were fasted for 24 h to empty the gastrointestinal tract, then force‐fed 150 g of the assigned diet. Excreta were quantitatively collected for 24 h. To minimize nitrogen volatilization, excreta were sprayed every 3 h with 5% boric acid solution. Samples were dried, ground, and analyzed for gross energy (GE) and nitrogen (N). Gross energy was measured by complete combustion in an adiabatic bomb calorimeter (model 1261; Parr Instrument Co., Moline, IL, USA), using benzoic acid as the calibration standard. Nitrogen was determined by the Kjeldahl method according to AOAC 968.06, and crude protein was calculated as *N* × 6.25.

Apparent metabolizable energy (AME), nitrogen‐corrected AME (AMEn), and nitrogen retention (NR) were calculated as follows:

$$\mathrm{AME}\;\left(\text{kcal}/\mathrm{kg}\right)=\left[\mathrm{GE}\;\text{intake}—\mathrm{GE}\;\text{excreta}\right]/\text{feed intake}$$

$$\text{AMEn}\;\left(\text{kcal}/\mathrm{kg}\right)=\mathrm{AME}—8.22\times \left[N\;\text{retained}\;\left(g\right)/\text{feed intake}\;\left(\mathrm{kg}\right)\right]$$

$$\mathrm{NR}\;\left(\%\right)=\left[\left(N\;\text{intake}—N\;\text{excreta}\right)/N\;\text{intake}\right]\times 100$$

### 2.8 Apparent Nutrient Digestibility

Apparent total‐tract digestibility of dry matter (DMD), organic matter (OMD), and crude protein (CPD) was determined from quantitative excreta collection during the metabolizable energy trial. Digestibility coefficients were calculated based on nutrient intake and excretion as follows:

$$\begin{array}{c} \text{Digestibility}\;\left(\%\right)=100\\ \times \left(\text{Nutrient intake}—\text{Nutrient excreted}\right)/\text{Nutrient intake} \end{array}$$

### 2.9 Digestive Enzyme Activity

After the metabolizable energy trial, the same selected birds (two per pen) were humanely slaughtered for sampling, and duodenal content was collected. Approximately 1 g of digesta was transferred into 10 mL phosphate buffer (0.05 M, pH 7.0), homogenized, and centrifuged at 12,000 rpm for 20 min using the centrifuge described above. Protease activity was assessed by a casein–Folin method using tyrosine as the reference standard; after incubation with 1% casein at 37°C, the reaction was stopped with 5% TCA, and absorbance was read at 660 nm on a SpectraMax 190 microplate reader. Amylase activity was determined by the DNS method based on reducing sugars released from starch, with absorbance read at 515.6 nm. Lipase activity was measured by titration using olive oil as substrate; liberated free fatty acids were quantified by titration with 0.05 M NaOH after incubation at 37°C.

### 2.10 Intestinal Microbial Enumeration

Intestinal content samples were serially diluted in sterile saline and plated on selective media for enumeration of total aerobic bacteria, *Escherichia coli* , and *Staphylococcus aureus*. Nutrient agar, eosin methylene blue agar (EMBA), and mannitol salt agar (MSA) (Merck KGaA, Darmstadt, Germany) were used for total aerobic bacteria, *E. coli* , and *S. aureus* , respectively. Plates were incubated at 37°C for 24–48 h in an IN55 microbiological incubator (Memmert GmbH + Co. KG, Schwabach, Germany), and results were expressed as colony‐forming units per gram (CFU/g). For statistical analysis, microbial counts were log10‐transformed to stabilize variances.

### 2.11 Statistical Analysis

Data were analyzed by one‐way analysis of variance using the MIXED procedure of SAS version 9.3 (SAS Institute Inc., Cary, NC, USA). The statistical model was Yij = μ + Ti + εij, where Yij is the observation for treatment i in replicate j, μ is the overall mean, Ti is the fixed effect of treatment, and εij is the residual error. Model residuals were examined before final inference to verify normality and homogeneity of variance. When the treatment effect was significant (*p* < 0.05), means were separated using Tukey's honestly significant difference (HSD) test (*α* = 0.05). Results are presented as treatment means with pooled SEM, and ANOVA test statistics are reported as F (5, 18) with the corresponding *p* value.

## 3 Results

### 3.1 Characterization of the Fermentate and Microcapsules

Fermentation monitoring indicated a stable process window (pH 5.10–5.18; temperature 32.75°C–34.97°C; moisture 72.83%–73.57%; Table S2). The resulting microcapsules had a product yield of 59.98% (w/w of total solids) with 30.47% moisture content and contained 37.07% crude protein and 26.97% ether extract on an as‐is basis (Table S1). Water activity (aw) ranged from 0.21 to 0.26 at 25°C; encapsulation efficiency (EE, protein/peptide‐based) was 91.96%, and particle size was standardized by sieving through a 180‐μm mesh (approximate D50 ≤ 180 μm) (Table S1). Total titratable acidity was 31.00 g lactic acid equivalents/L, and soluble protein/peptides were 3.50 mg/mL (undiluted; Bradford, DF = 10) (Table S2). VFAs (acetate/propionate/butyrate) were not detected by GC‐FID (below lab‐reported LOD/LOQ: acetate 1.63, propionate 0.164, butyrate 0.067; internal standard used).

Metabolizable energy and nitrogen retention are summarized in Table 3. Dietary inclusion of MFCWE affected AME (F (5, 18) = 5.44, *p* = 0.003) and AMEn (F (5, 18) = 4.58, *p* = 0.007), with the highest values observed at 1.5%–2.0% inclusion. Relative to T‐, 1.5% MFCWE increased AME by 2.1% and AMEn by 2.0%; relative to T+, the corresponding increases were 1.7% and 2.2%. Nitrogen retention was also affected (F (5, 18) = 8.72, *p* < 0.001) and was maximized at 1.5% MFCWE, representing a 4.7% increase relative to T−.

**TABLE 3:** Apparent metabolizable energy (AME), nitrogen‐corrected AME (AMEn), and nitrogen retention (NR) of broilers fed experimental diets.

Treatment	AME (kcal/kg)	AMEn (kcal/kg)	NR (%)
T−	2751^ab^	2731^ab^	66.83^b^
T+	2763^ab^	2724^ab^	67.99^a^
T0.5%	2705^b^	2685^b^	65.95^b^
T1%	2757^ab^	2736^ab^	68.05^a^
T1.5%	2810^a^	2785^a^	69.96^a^
T2%	2804^a^	2781^a^	65.42^b^
SEM	16.51	17.59	0.56
*p* value	0.003	0.007	< 0.001

*Note:* Values are treatment means (pen as the experimental unit; *n* = 4 pens per treatment). SEM, pooled standard error of the mean. Within a column, means with different superscript letters differ significantly (Tukey's HSD, *p* < 0.05).

Apparent nutrient digestibility results are presented in Table 4. Treatment affected DMD (F (5, 18) = 69.25, *p* < 0.001) and OMD (F (5, 18) = 85.08, *p* < 0.001); both were improved at 1.5% MFCWE and were comparable to the antibiotic control. Relative to T−, 1.5% MFCWE increased DMD by 8.6% and OMD by 7.6%. Crude protein digestibility was also affected (F (5, 18) = 5.61, *p* = 0.003), with the lowest value observed at 2.0% inclusion.

**TABLE 4:** Apparent digestibility of dry matter (DMD), organic matter (OMD), and crude protein (CPD) of broilers fed experimental diets.

Treatment	DMD (%)	OMD (%)	CPD (%)
T−	57.05^b^	59.84^b^	64.40^ab^
T+	61.06^a^	64.89^a^	70.62^a^
T0.5%	51.95^c^	55.40^c^	66.05^ab^
T1%	55.27^b^	58.39^b^	64.40^ab^
T1.5%	61.93^a^	64.39^a^	66.22^ab^
T2%	55.96^b^	58.75^b^	59.74^b^
SEM	0.45	0.40	1.49
*p* value	< 0.001	< 0.001	0.003

*Note:* Values are treatment means (pen as the experimental unit; *n* = 4 pens per treatment). SEM, pooled standard error of the mean. Within a column, means with different superscript letters differ significantly (Tukey's HSD, *p* < 0.05).

Digestive enzyme activity is shown in Table 5. MFCWE increased amylase activity (F (5, 18) = 5.06, *p* = 0.005), whereas protease (*p* = 0.190) and lipase (*p* = 0.252) were not significantly affected. At 1.5% inclusion, amylase activity was 34.5% higher than T− and only 1.9% lower than T+.

**TABLE 5:** Digestive enzyme activity in the duodenal content of broilers fed experimental diets.

Treatment	Protease (U/mL)	Amylase (U/mL)	Lipase (U/mL)
T−	0.249	20.69^b^	2.00
T+	0.262	28.36^a^	3.31
T0.5%	0.266	23.67^ab^	2.38
T1%	0.273	25.67^ab^	3.44
T1.5%	0.263	27.83^a^	3.19
T2%	0.266	21.46^b^	4.25
SEM	0.006	1.43	0.66
*p* value	0.190	0.005	0.252

*Note:* Values are treatment means (pen as the experimental unit; *n* = 4 pens per treatment). SEM, pooled standard error of the mean. Within a column, means with different superscript letters differ significantly (Tukey's HSD, *p* < 0.05).

Intestinal microbial counts (log10 CFU/g) are presented in Table 6. MFCWE reduced *S. aureus* counts (F (5, 18) = 5.43, *p* = 0.003), whereas total aerobic bacteria (*p* = 0.363) and *E. coli* (*p* = 0.998) were not affected. At 1.5% inclusion, *S. aureus* was 16.6% lower than T− and 11.8% lower than T+.

**TABLE 6:** Intestinal microbial counts (log10 CFU/g) of broilers fed experimental diets.

Treatment	Total aerobic bacteria	*E. coli*	*S. aureus*
T−	10.793	5.333	5.219^a^
T+	10.555	5.320	4.939^a^
T0.5%	10.671	5.308	5.079^a^
T1%	10.517	5.335	4.785^ab^
T1.5%	10.623	5.298	4.354^b^
T2%	10.525	5.311	4.834^ab^
SEM	0.098	0.064	0.128
*p* value	0.363	0.998	0.003

*Note:* Values are treatment means (pen as the experimental unit; *n* = 4 pens per treatment) and were analyzed on log10‐transformed counts. SEM, pooled standard error of the mean. Within a column, means with different superscript letters differ significantly (Tukey's HSD, *p* < 0.05).

## 4 Discussion

The present findings indicate that MFCWE modulated nutrient utilization and gut functional indicators in broilers, with the most consistent responses at 1.5% inclusion. A plausible explanation is that fermentation partially hydrolyzed the catfish byproduct matrix into more accessible low‐molecular‐weight nutrients and metabolites, whereas microencapsulation helped protect these labile fractions during drying, storage, and feed mixing. In that context, the higher AME/AMEn observed at 1.5%–2.0% inclusion likely reflects both improved substrate availability and more stable delivery of fermentation‐derived bioactives to the digestive tract (Beckman et al. 2024; Cristobal et al. 2025; Eshtejarani et al. 2024).

The improvement in dry matter and organic matter digestibility at 1.5% MFCWE suggests that the bioactive fraction of the fermented extract supported gastrointestinal function beyond simple nutrient supply. Fermentation can generate small peptides, free amino acids, and acidifying metabolites that enhance digestibility, while microencapsulation may improve the uniformity of intake and reduce premature oxidation of the active fraction. This interpretation is consistent with reports that postbiotic or fermented feed preparations can improve digestive efficiency and barrier function in poultry and other monogastrics (Dastar et al. 2025; Saleh et al. 2025; Woyengo et al. 2025).

Digestive enzyme activity is a sensitive marker of pancreatic stimulation and intestinal functional adaptation. The marked increase in amylase activity at 1.5% MFCWE may indicate improved carbohydrate digestion and a more favorable luminal environment for endogenous enzyme function. Because the pancreas is the principal source of amylase, lipase, and protease secretion in poultry, this response may reflect indirect stimulation of digestive physiology by fermentation‐derived nutrients and metabolites rather than a direct enzyme‐supplement effect alone (Karunaratne et al. 2021; Lushchak 2025; Rubio‐Cervantes et al. 2025; Zaworski et al. 2024).

Microbial data showed a reduction in *S. aureus* without detectable changes in total aerobic bacteria or *E. coli* , suggesting a selective inhibitory effect rather than broad microbial suppression. This pattern is biologically plausible because fermentation‐derived acids and peptide‐like antimicrobials may be more active against Gram‐positive opportunists, while microencapsulation can help preserve such metabolites until feed delivery and digestion. Similar selective responses have been reported for microbial‐derived or encapsulated functional additives in poultry systems (Pipová et al. 2025; Shams Shargh et al. 2025; Tan et al. 2024; Xu et al. 2024; Zha et al. 2025).

Taken together, these results support the concept that microencapsulation can facilitate the valorization of fermented fish‐processing byproducts into more stable, functional feed additives, thereby extending the relevance of the study beyond broiler production to food‐system sustainability and circular bioeconomy applications. Limitations of the present work include the use of culture‐based microbial indicators rather than high‐resolution community profiling and the absence of pathogen‐challenge validation. In addition, VFAs (acetate/propionate/butyrate) were below the laboratory‐reported LOD/LOQ by GC‐FID, and therefore, the mechanistic interpretation focused on pH, TTA, and soluble protein/peptides. Future work should integrate challenge trials (e.g., Salmonella or necrotic enteritis/coccidiosis models as commonly used in postbiotic research), shelf‐life/oxidative stability testing of microcapsules, targeted metabolite profiling (including method‐validated VFA quantification where feasible), and next‐generation sequencing to link metabolite signatures with microbiota shifts and performance outcomes (Guo et al. 2025; Otto et al. 2025).

### 4.1 Practical Implications and Future Recommendations

Translate the current efficacy signals (improved AME/AMEn and digestibility; selective reduction of *S. aureus* indicators) into field‐relevant outcomes by conducting longer grow‐out trials that include growth performance, feed conversion ratio, carcass yield, and meat quality under commercial‐like conditions.

Validate robustness and shelf‐life of the microcapsules by monitoring water activity–moisture dynamics, oxidative stability (e.g., peroxide value/TBARS where relevant), and bioactive retention (protein/peptide‐based EE) during storage and after feed processing (e.g., pelleting temperature/time).

## 5 Conclusion

Microencapsulated fermented catfish waste extract improved energy utilization, nutrient digestibility, intestinal amylase activity, and suppression of *Staphylococcus aureus* in broiler chickens, with 1.5% inclusion producing the most consistent overall response. These results support MFCWE as a promising alternative to antibiotic growth promoters and as a value‐added use of catfish‐processing waste, although the findings should be interpreted in light of the culture‐based microbial assessment and the absence of challenge‐model validation.

## Author Contributions

**Rahmad Fany Ramdhan:** investigation, validation, visualization, writing – review and editing, software, project administration, supervision, resources. **Kiki Haetami:** investigation, funding acquisition, writing – original draft, validation, visualization, writing – review and editing, formal analysis, project administration, data curation, resources. **Abun Abun:** conceptualization, investigation, funding acquisition, writing – original draft, methodology, validation, writing – review and editing, software, data curation, supervision, resources.

## Funding

This work was supported by 0419/C3/DT.05.00/2025, 093/C3/DT.05.00/PL/2025, 1507/UN6.3.1/PT.00/2025, UNP21.

## Ethics Statement

All animal procedures were approved by the Research Ethics Committee of Universitas Padjadjaran, Bandung, Indonesia (Protocol No. 231/UN6.KEP/EC/2025; approved on 14 March 2025) and complied with institutional guidelines for the care and use of animals in research. This study is reported in accordance with ARRIVE 2.0 (Essential 10).

## Conflicts of Interest

The authors declare no conflicts of interest.

## Supporting information


**Table S1:** Physicochemical characterization of MFCWE microcapsules (product documentation).
**Table S2:** Fermentation metabolite indicators of the cell‐free fermentate (process characterization).

## Data Availability

The data that support the findings of this study are available from the corresponding author upon reasonable request. Key outcomes are provided in Tables 1, 2, 3, 4, 5, 6.
