# Supplementary material for: Effect of Microencapsulated Fermented Catfish Waste Extract on Apparent Metabolizable Energy, Nutrient Digestibility, Digestive Enzyme Activity, and Intestinal Microbiota of Broiler Chickens
Source: Food Sci Nutr. 2026 Jun 10;14(6):e72012. doi: 10.1002/fsn3.72012 (PMC13251437; doi:10.1002/fsn3.72012)
Supplement: Supplementary file 1 — Table S1: Physicochemical characterization of MFCWE microcapsules (product documentation). Table S2: Fermentation metabolite indicators of the cell‐free fermentate (process characterization). [file FSN3-14-e72012-s001.docx]

**Supporting Information**

*Effect of microencapsulated fermented catfish waste extract on apparent metabolizable energy, nutrient digestibility, digestive enzyme activity, and intestinal microbiota of broiler chickens*

**Abun Abun, Kiki Haetami, and Rahmad Fany Ramdhan**

**Table S1.** Physicochemical characterization of MFCWE microcapsules (product documentation).

Abbreviations: MFCWE, microencapsulated fermented catfish waste extract; EE, encapsulation efficiency; aw, water activity.

| **Parameter** | **Unit** | **Result** |
| --- | --- | --- |
| Yield | % (w/w) | 59.98 |
| Moisture content | % | 30.47 |
| Water activity | aw | 0.21–0.26 (25°C) |
| Encapsulation efficiency | % | 91.96 |
| Particle size (D50) | µm | ≤180 (sieve cut-off) |
| Crude protein | % (as-is) | 37.07 |
| Ether extract | % (as-is) | 26.97 |

**Table S2.** Fermentation metabolite indicators of the cell-free fermentate (process characterization).

Abbreviations: TTA, total titratable acidity; VFA, volatile fatty acid; GC-FID, gas chromatography-flame ionization detection; LOD, limit of detection; LOQ, limit of quantification.

| **Indicator** | **Unit** | **Result** |
| --- | --- | --- |
| pH (during fermentation) | — | 5.10–5.18 |
| Temperature (during fermentation) | °C | 32.75–34.97 |
| Moisture (during fermentation) | % | 72.83–73.57 |
| Total titratable acidity | g lactic acid/L | 31.00 |
| Soluble protein/peptides | mg/mL | 3.50 |
| VFAs (acetate/propionate/butyrate) | mM (mmol/L) (GC-FID) | Not detected (below lab-reported LOD/LOQ; acetate 1.63, propionate 0.16, butyrate 0.067 mM (mmol/L); internal standard used) |
